# Transcriptome profiling of Arabian horse blood during training regimens

**DOI:** 10.1186/s12863-017-0499-1

**Published:** 2017-04-05

**Authors:** Katarzyna Ropka-Molik, Monika Stefaniuk-Szmukier, Kacper Żukowski, Katarzyna Piórkowska, Artur Gurgul, Monika Bugno-Poniewierska

**Affiliations:** 1 Department of Genomics and Animal Molecular Biology, National Research Institute of Animal Production, Balice, Poland; 2 Department of Horse Breeding, Institute of Animal Science, University of Agriculture in Cracow, Kracow, Poland; 3 Department of Animal Genetics and Breeding, National Research Institute of Animal Production, Balice, Poland

**Keywords:** Arabian horse, Training, Blood transcriptome, RNA-seq, Exercise adaptation

## Abstract

**Background:**

Arabian horses are believed to be one of the oldest and most influential horse breeds in the world. Blood is the main tissue involved in maintaining body homeostasis, and it is considered a marker of the processes taking place in the other tissues. Thus, the aim of our study was to identify the genetic basis of changes occurring in the blood of Arabian horses subjected to a training regimen and to compare the global gene expression profiles between different training periods (T_1_: after a slow canter phase that is considered a conditioning phase, T_2_: after an intense gallop phase, and T_3_: at the end of the racing season) and between trained and untrained horses (T_0_). RNA sequencing was performed on 37 samples with a 75-bp single-end run on a HiScanSQ platform (Illumina), and differentially expressed genes (DEGs) were identified based on DESeq2 (v1.11.25) software.

**Results:**

An increase in the number of DEGs between subsequent training periods was observed, and the highest amount of DEGs (440) was detected between untrained horses (T_0_) and horses at the end of the racing season (T_3_). The comparisons of the T_2_ vs. T_3_ transcriptomes and the T_0_ vs. T_3_ transcriptomes showed a significant gain of up-regulated genes during long-term exercise (up-regulation of 266 and 389 DEGs in the T_3_ period compared to T_2_ and T_0_, respectively). Forty differentially expressed genes were detected between the T_1_ and T_2_ periods, and 296 between T_2_ and T_3_. Functional annotation showed that the most abundant genes up-regulated in exercise were involved in pathways regulating cell cycle (PI3K-Akt signalling pathway), cell communication (cAMP-dependent pathway), proliferation, differentiation and apoptosis, as well as immunity processes (Jak-STAT signalling pathway).

**Conclusions:**

We investigated whether training causes permanent transcriptome changes in horse blood as a reflection of adaptation to conditioning and the maintenance of fitness to compete in flat races. The present study identified the overrepresented molecular pathways and genes that are essential for maintaining body homeostasis during long-term exercise in Arabian horses. Selected DEGs should be further investigated as markers that are potentially associated with racing performance in Arabian horses.

**Electronic supplementary material:**

The online version of this article (doi:10.1186/s12863-017-0499-1) contains supplementary material, which is available to authorized users.

## Background

It is well established that intensive training initiates long-term adaptation processes that are involved in the establishment of a new body homeostasis. During exercise, the oxygen demand significantly increases, and thus, one of the most important adaptations to training is optimization of O_2_ transport in the entire organism by both improvements in vascular endothelial function and increases in mitochondrial function [1, 2]. On the other hand, an imbalance between exercise and the recovery process can result in negative consequences, such as underperformance and a progressive fatigue called overtraining syndrome [3, 4]. It seems to be important to distinguish the signs of overtraining from the effects of balanced exercise on the molecular level in order to design molecular tools for training optimization. To date, only the protein levels of alpha-1 antitrypsin has been considered as a marker of overtraining in horses based on protein analysis during normal and intensive training [5].

In humans, several studies emphasized the need for identifying a possible genetic predisposition to athletic performance and for detecting the molecular pathways involved in overtraining syndrome [4]. The horse is considered as an excellent model for analysing the changes that occur in response to exercise-induced stress. In recent decades, huge improvements in molecular genetics methods have enabled genome-wide searches for genes associated with important phenotypic features. Due to the high profitability of sport and racing horses, a major area of research is to identify the genetic basis of athletic performance in Arabian horses.

The first analysis of changes in muscle gene expression profiles during exercise adaptation was performed in Thoroughbreds using cDNA microarrays [6]. In 2010, the research performed by McGivney et al. [7] using RNA sequencing (Next-Generation technology) confirmed the differential expression of 92 transcripts in equine muscle tissues before and after long-term exercise. Among the genes whose expression decreased after exercise, the authors identified the myostatin gene (*MSTN*). The strong association of MSTN with the best race distances and athletic performance in Thoroughbreds was later confirmed by other studies [8, 9]. The comparative transcriptome analysis of blood and muscle cells in six Thoroughbred horses showed the common expression of over 11,300 genes in both tissues, which constituted over 60% of all genes transcribed in muscle [10]. Furthermore, the authors confirmed the significant effects of intense exercise on the transcriptome profiles of both tissues [10].

The racing performance traits of Thoroughbred horses (TB) have been widely described. On the other hand, the exact mechanism that occurs in the blood and muscles of Arabian horses during exercise in relation to stamina and performance is still not well understood. In purebred Arabians, a main selection criteria is racetrack performance. Young (3- to 4-year-old) horses compete in races, and this age has a significant effect on their development. After their career on the race track is finished, the predisposed horses are prepared for endurance rides.

Capomaccio et al. [11], who analysed whole transcriptome profiles of blood in Arabian horses, pinpointed the biological processes that are potentially related to exercise-induced stress and identified groups of genes that are related to inflammation, immune interaction or cell signalling. Such research is intended to broaden our knowledge of the molecular mechanisms associated with adaptation to exercise in order to select the most suitable training schedules for obtaining better performance as well as maintaining animal health [12].

It has been established that repeated rounds of exercise lead to new basal levels of gene expression in resting muscles [13]. Blood is the main tissue involved in maintaining body homeostasis, and it is considered a marker of the processes taking place in the other tissues. Thus, the aim of our study was to identify the genetic basis of changes occurring in the blood of Arabian horses during their training regimen. We investigated whether the training period causes permanent transcriptome changes that are involved in the horse’s adaptation to conditioning and maintenance of its fitness to compete in flat races.

## Methods

### Animals

The profiling of blood transcriptomes was performed for 12 Arabian horses (3 years old) that were introduced to the training centre (to prepare for flat racing). Blood samples were collected at 3 different time points during the training procedure: after the slow canter phase, which is considered a conditioning phase (March; T_1_); after an intense gallop phase (May, trained horses before the racing season; T_2_); and at the end of the racing season (October; T_3_). The blood transcriptomes of 6 untrained Arabian horses (2.5 years old; T_0_) were also analysed. It was not possible to collect blood samples from all of the same horses in the control group as a training group (some horses sampled as untrained were not introduced to the training centre). The analysed group of animals included the same number of mares and stallions in order to avoid a gender effect.

To identify genes with the greatest effect on adaptation to long-term exercise, we compared transcriptomes starting from period T_1_ (conditioning phase) to T_3_ (end of training after the racing season): T_1_ → T_2_ → T_3_ (training comprised one year). Additionally, we compared transcriptome profiles between the T_3_ (trained horses) and T_0_ (untrained horses, control group) periods in order to detect potential training-induced modifications of gene expression: T_1_ → T_2_ → T_3_ → T_0_.

All animals were healthy (they were under veterinary supervision) and successfully performed on the racetrack during the racing season. In each period, samples were collected at the same time of the day (2 h after morning feeding and before training to avoid disruption of the training routine) and according to the same procedure for each horse. The exact training schedule and the time points of sample collection are precisely described in the Additional file 1: Figure S1. All horses were maintained in one stud, which was owned by one breeder. In addition, they were introduced in the Polish Arabian Stud Book (PASB) while also belonging to the pure Polish line of known origin since the late 1700s. They were also maintained under the same environmental and feeding conditions (fed a hay- and oat-based diet that was supplemented with a commercial feed mixture according to the stage of the training cycle).

The protocol was approved by the Animal Care and Use Committee of the Institute of Pharmacology, Polish Academy of Sciences in Kraków (no. 1173/2015). Owners consent for their horses’ inclusion in the study.

### Transcriptome sequencing

In total, 42 blood samples were collected from the jugular vein into Tempus™ Blood RNA Tubes (Ambion, Life Technologies) and stored at −20 °C. Total RNA was isolated using the MagMAX™-96 Total RNA Isolation Kit (Ambion, Life Technologies) according to the manufacturer’s protocol. The quality and quantity of obtained RNA were examined using NanoDrop 2000 (Thermo Scientific; Wilmington, USA) and TapeStation 2200 instruments with Agilent RNA ScreenTape (Agilent, Perlan Technologies). The RNA samples with RIN (RNA integrity number) values above 8.5 were used for further analysis. Five samples were removed from RNA-seq analysis due to the exclusion of horses from the training cycle for various reasons (lameness or poor metabolic performance).

The cDNA libraries were prepared from 400 ng of total RNA with TruSeq RNA Sample Prep Kit v2 kit (Illumina) according to the protocol. The quality and quantity of the obtained libraries were established using Qubit 2.0 (Invitrogen, Life Technologies) and TapeStation 2200 (D1000 ScreenTape; Agilent). RNA sequencing was performed with a 75-bp single-end run on a HiScanSQ platform (Illumina) using TruSeq SR Cluster Kit v3- CBOT-HS and TruSeq SBS Kit v 3 - HS chemistry (Illumina). Indexed samples were pooled into two groups and loaded into 3–4 lanes on two flow-cells, which allowed for obtaining at least 3 technical replications per library.

### Data analysis

Quality control (QC) of the raw sequence data was conducted using FastQC software (v0.11.4). The obtained QC results were used as an indicator to remove adapter sequences, reads shorter than 36 bp and/or reads with a quality score lower than 20 (Flexbar software v2.5) [14]. The quantification of transcript abundance was conducted using RSEM software (v1.2.22) [15] supported by STAR aligner software (STAR_2.4.2a) [16]. The raw reads were aligned to the *Equus caballus* genome (assembly Equus_caballus.EquCab2.83) as a reference, while the whole procedure was followed by alignment parameter evaluation using ENCODE3's STAR-RSEM pipeline. To detect the differentially expressed genes (DEGs), Bioconductor/R-project package (R version 3.3.1) was used with DESeq2 (1.12.4) (SummarizedExperiment_1.2.3; Biobase_2.32.0; GenomicRanges_1.24.2) (adjusted p-value was calculated using the Benjamini/Hochberg method) [17].

Gene Ontology (GO) annotation was performed for DEGs (fold change ≥ |1.3|; adjusted *p*-value < 0.05) using Panther software [18]. Because variability in the fold change (FC) of a gene is inversely related to its expression level [19], in the present study, DEGs with FC > 1.3 (*p*-value < 0.05) were considered significant in order to avoid overlooking the exercise–induced modifications of highly expressed genes. GO annotations were performed using the latest version of the *Equus caballus* reference, which includes 20,384 annotated genes. The p-value in enrichment tests was determined based on the Mann–Whitney *U* Test (Wilcoxon Rank-Sum Test) with a Bonferroni correction. The online DAVID software and the Kyoto Encyclopedia of Genes and Genomes (KEGG) with KEGG mapper – search pathway tools was applied for pathway detection and analysis. The significantly enriched pathways were identified based on the p-values obtained from a Fisher Exact test.

### qPCR validation

The evaluation of mRNA levels of eight selected DEGs was performed by a real-time qPCR method. Genes selected for validation were identified as significantly differentially expressed in at least two comparisons between different training periods.

cDNA was synthesized for all analysed samples from 500 ng of total RNA with the use of a TranscriptME RNA kit (Blirt, Poland), according to manufacturer’s protocol. The determination of gene expression was performed on a 7500 Real-Time PCR System using AmpliQ 5x HOT EvaGreen® qPCR Mix Plus (ROX) (Novazym, Poland) in three technical replicates for each sample. Two different genes were used as endogenous controls: *GAPDH* (glyceraldehyde 3-phosphate dehydrogenase) and *SDHA* (succinate dehydrogenase complex subunit A) [20]. Primers for the target and control genes were designed using version 4.0.0 of Primer3 software based on the reference sequences annotated in the Ensembl database (Additional file 2: Table S1). For all genes except *P2RY14*, PCR products spanned two exons. The efficiency of the qPCR reactions was defined using the standard curve method, and mRNA abundance was calculated according to the ∆∆Ct method.

Pearson’s correlation was used to compare the normalized read counts and qPCR data. A one-way ANOVA with Duncan’s post hoc test was used to estimate the significance of differences in gene expression between training periods.

## Results

### Sequencing data

In total, 914.7 million raw reads were generated for all samples, ranging from 16.4 to 27.7 M per sample. On average, individual samples yielded 24.7 M (±3.8) reads. After quality trimming and filtration, 898 M high-quality reads (98.2% of all reads) were used for mapping. According to the STAR-RSEM pipeline, there were 7.8 – 28.2 M (19.7 M or 81%, on average) uniquely aligned reads per sample. Detailed statistics on the number of analysed reads and achieved mapping efficiency are presented in Additional file 3: Table S2. Approximately 81% of raw reads were mapped to the EquCab2.0 assembly, 51% of which matched annotated exonic regions, 16% matched intronic regions, and 33% matched intergenic regions. The analysis was submitted to the NCBI Gene Expression Omnibus (GEO) functional genomics data repository and assigned GEO accession number GSE83404 (http://www.ncbi.nlm.nih.gov/geo/query/acc.cgi?acc=GSE83404).

### Exercise-regulated differentially expressed genes

In the present research, the blood transcriptome profiles between horses in successive training stages and a control group were compared as follows: T_1_:T_2_, T_2_:T_3_, and T_0:_T_3_. (T_1_ → T_2_ → T_3_ → T_0_)_._ An increase in the number of DEGs (fold change at of least ±1.3; adjusted p-value < 0.05) between subsequent training periods was observed, and the highest amount of DEGs (440) was detected between untrained horses (T_0_) and horses at the end of the racing season (T_3_) (Table 1). The comparisons of the transcriptomes T_2_ vs. T_3_ and T_0_ vs. T_3_ showed a significant gain in up-regulated genes during long-term exercise (up-regulation of 266 and 389 DEGs in T_3_ period compared to T_2_ and T_0_, respectively). Forty differentially expressed genes were detected between the T_1_ and T_2_ periods, and 296 between T_2_ and T_3_.

**Table 1:** The number of identified differentially expressed genes (DEGs) between different training periods

Comparison	T_1_:T_2_		T_2_:T_3_		T_0_:T_3_	
	Down	Up	Down	Up	Down	Up
Annotated gene	34		269		389	
	15	19	19	250	28	361
Novel gene	6		27		51	
	–	6	11	16	23	28
Total DEGs	40		296		440	

Furthermore, when comparing the transcriptomes in the T_0_ vs. T_3_ exercise stages, 51 novel exercise-regulated genes (annotated as novel genes according to the Ensemble database) were detected: 23 were up-regulated and 28 were down-regulated, which accounted for approximately 11% of all identified DEGs. Due to the high number of uncharacterized DEGs, the functional classification of orthologues was performed using Panther software. The analysis showed that some of the gene orthologues classified as novel corresponded to protein families and/or protein classes (Additional file 4: Table S3). The most overrepresented functional group included a panel of orthologous genes related to binding activity (molecular function, GO:0005488; e.g., *TBL1X, TAF1, SRCAP, AKAP11, POM121C, RBM27*) and biological regulation (biological process, GO:0065007; *TBL1X, TAF1, SRCAP, AKAP11* and LOC100062658 orthologs). Some of the genes identified as novel were differentially expressed in two out of three comparisons (T_2_:T_3_ and T_0_:T_3_)_,_ which suggested that selected genes can play an important role during the adaptation process to exercise (orthologues of *TBL1X, RBM27, GOPC, KMT2C, LY5, PRR14L, TRAPPC10, LOC100062658*) (Additional file 4: Table S3).

We also compared the sets of significant DEGs for T_1_ vs. T_2_, T_2_ vs. T_3_ and T_0_ vs. T_3_, and we detected several genes whose transcription was down- or up-regulated during at least two training periods (Table 2). According to their GO annotations, the identified genes play a role in the regulation of biological processes (GO:0050789; NC*OA2, P2RY14, MBTD1, ZNF618, NFAT5*), homeostatic processes (GO:0042592; *ATP13A3*) and metabolic processes (nucleobase-containing compound metabolic process, GO:0006139; *NCOA2, P2RY14, MBTD1, ZNF618, NFAT5, WDR7*).

**Table 2:** List of exercise regulated (down or up-regulated) genes during at least two training periods

Training periods	Gene name	Ensembl Accession number	T_1_:T_2_		T_2_:T_3_		T_0_:T_3_	
			FC	adj p	FC	adj p	FC	adj p
Genes up-regulated								
1	*ACVR2A*	ENSECAG00000000138	1.56	0.01	1.55	0,003	ns	
2	*ZAB2*	ENSECAG00000019355	1.41	0.05	1.42	0.017	ns	
3	*P2RY14*	ENSECAG00000001554	1.54	0.05	1.69	0.009	1.59	0.05
4	*WDR7*	ENSECAG00000007050	1.37	0.006	1.60	0.01	1.60	0.005
5	*AGPAT1*	ENSECAG00000016106	1.55	0.011	1.60	0.02	1.52	0.02
6	*FOXN2*	ENSECAG00000008390	ns		1.80	0.007	2.01	8.78E-06
7	*LARP4*	ENSECAG00000012479	ns		1.54	0.013	1.65	0.005
8	*LPGAT1*	ENSECAG00000013187	ns		1.39	0.01	1.46	0.02
9	*RICTOR*	ENSECAG00000000308	ns		1.74	0.002	1.91	0.0002
10	*ATP13A3*	ENSECAG00000006749	ns		1.65	0.0009	1.89	0.0005
11	*HIPK1*	ENSECAG00000015193	ns		1.61	0.013	1.92	0.0001
12	*NFAT5*	ENSECAG00000010266	ns		1.66	0.016	1.97	0.0008
13	*ZNF618*	ENSECAG00000020621	ns		1.66	0.003	1.87	0.0006
14	*GALNT7*	ENSECAG00000010243	ns		1.61	0.009	1.57	0.011
15	*TMED5*	ENSECAG00000016426	ns		1.51	0.01	1.53	0.003
16	*MBTD1*	ENSECAG00000022700	ns		1.65	0.009	1.61	0.005
17	*NCOA2*	ENSECAG00000008394	ns		1.41	0.05	1.61	0.0003
Genes down-regulated								
1	*GTSF1*	ENSECAG00000013618	ns		−1.55	0.0005	−1.48	0.034
2	*novel*	ENSECAG00000015782	ns		−1.83	0.001	−1.77	0.012
3	*novel*	ENSECAG00000017143	ns		−1.49	0.03	−1.66	0.02
4	*ANKZF1*	ENSECAG00000016473	0.5	0.05	−1.37	0.003	ns	
5	*C7orf50*	ENSECAG00000022930	−1.39	0.007	−1.39	0.04	ns	
6	*GZMH*	ENSECAG00000008322	−1.57	0.05	−1.46	0.04	ns	
7	*SYCE1*	ENSECAG00000012421	−1.43	0.01	−1.83	0.001	ns	
8	*CRYGS*	ENSECAG00000002645	−1.39	0.007	−1.43	0.004	ns	
9	*C12orf43*	ENSECAG00000014885	−1.42	0.05	ns		−1.36	0.04

*FC* fold change, *ns* not significant

### Functional annotation and identified pathways

Our results showed that the highest number of identified genes encoded transcription factors, nucleic acid binding proteins and G-protein modulators, which were mainly transcriptionally activated at the last training phase (T_3_) (Table 3). Moreover, in the T_3_ period, the identified DEGs represented genes encoding cytoskeletal proteins, including actin cytoskeletal proteins and kinases. In turn, at the beginning of the training cycle (T_1_), the differential expression of genes related to immune response (defence proteins, cytokine receptors) and genes encoding hydrolase were detected (Table 3).

**Table 3:** The mostly affected genes encoding the most overrepresented protein classes during training regime in Arabian horses

	N	*P*	Gene	N	*P*	Gene	N	*P*	Gene
Training periods	T_1_ vs T_2_			T_2_ vs T_3_			T_0_ vs T_3_		
transcription factors (PC00218)	6	ns	*QKI; PPP1R37; PHTF2; MBTD1; FOXS1; ZEB2;*	34	0.0001	*AHR; GNPTAB; IKZF1; NCOA1; NFKBID; MBTD1; NFAT5; PDLIM5; RBBP8; RREB1*	46	0.006	*NFATC1;NCOR2; FOXJ2;NFAT5;SATB1;NCOA1;SKI;QKI;AFF4*
nucleic acid binding (PC00171)	13	ns	*LARP4; MBTD1; WDR7; RNASE1; OAS1; FOXS1*	42	0.003	*MDM4; ZBTB38; ACAP2; TOP3B; BDP1; WDR7; ATM; LARP4; SATB1*	74	0.0001	*CHD2; RREB1; TRA2A; MDM4; ZBTB38; SETX; ACAP2; NCOR2; WDR7; PRRC2C;*
G-protein modulator (PC00022)	2	ns	*P2RY14; CYSLTR1*	10	0.004	*CCRL2; P2RY14; EVI5; GNB4;*	16	0.02	*S1PR4;P2RY14;ACAP2;APPL1*
cytoskeletal protein (PC00085)							28	0.004	*CAMK4; MYO5A; DYNC1LI2; ACTR2; LMNB2; MSN; ASPM; SEPT11; MYH9; MYO9A; SYNE3*
actin family cytoskeletal protein (PC00041)							15	0.02	*MYO5A; ACTR2; KLHL6; MSN; SPTBN1; MYO9A; ASPM; KLHL11; WASF2; SYNE3; MYH9*
kinase (PC00137)	3	0.003	*ACVR2A, MAPK3, CASK*	17	0.0028	*PIK3CG; EEF2K; ACVR1B; ATM; MAP3K7; ACVR2A; WNK1; HIPK3; STK17B; LRRK2; HIPK1*	19	0.002	*PIK3CG; CAMK4; PANK3; MAP3K7; EEF2K; DYRK2; ATM; AURKC; HIPK3; HIPK1*
TGF-beta receptor (PC00035)	1	0.008	*ACVR2A*	1	0.0008	*ACVR1B; ACVR2A;*			
defense/immunity protein (PC00090)	5	0.001	*CD1C; C2; OAS1; ENSECAG00000012920; ENSECAG00000000325*	3	0.001	*TXLNG; IL6ST; CCRL2;*	4	0.003	*TXLNA;TXLNG; NOTCH1;NOTCH2;*
cytokine receptor (PC00084)	5	0.004	*ACVR2A; CD1C; ENSECAG00000012920; ENSECAG00000000325*	4	0.01	*CCRL2; ACVR2A; ACVR1B; IL6ST*			
hydrolase (PC00121)	5	0.002	*RNASE1; C2; WDR7; MX1; CHI3L1;*	8	0.01	*EVI5; ATP11C; NUDT17; ATP13A3; TPP1; GLS; PADI6; GNB4;*			

*N* number of genes detected

**Fig. 1:**
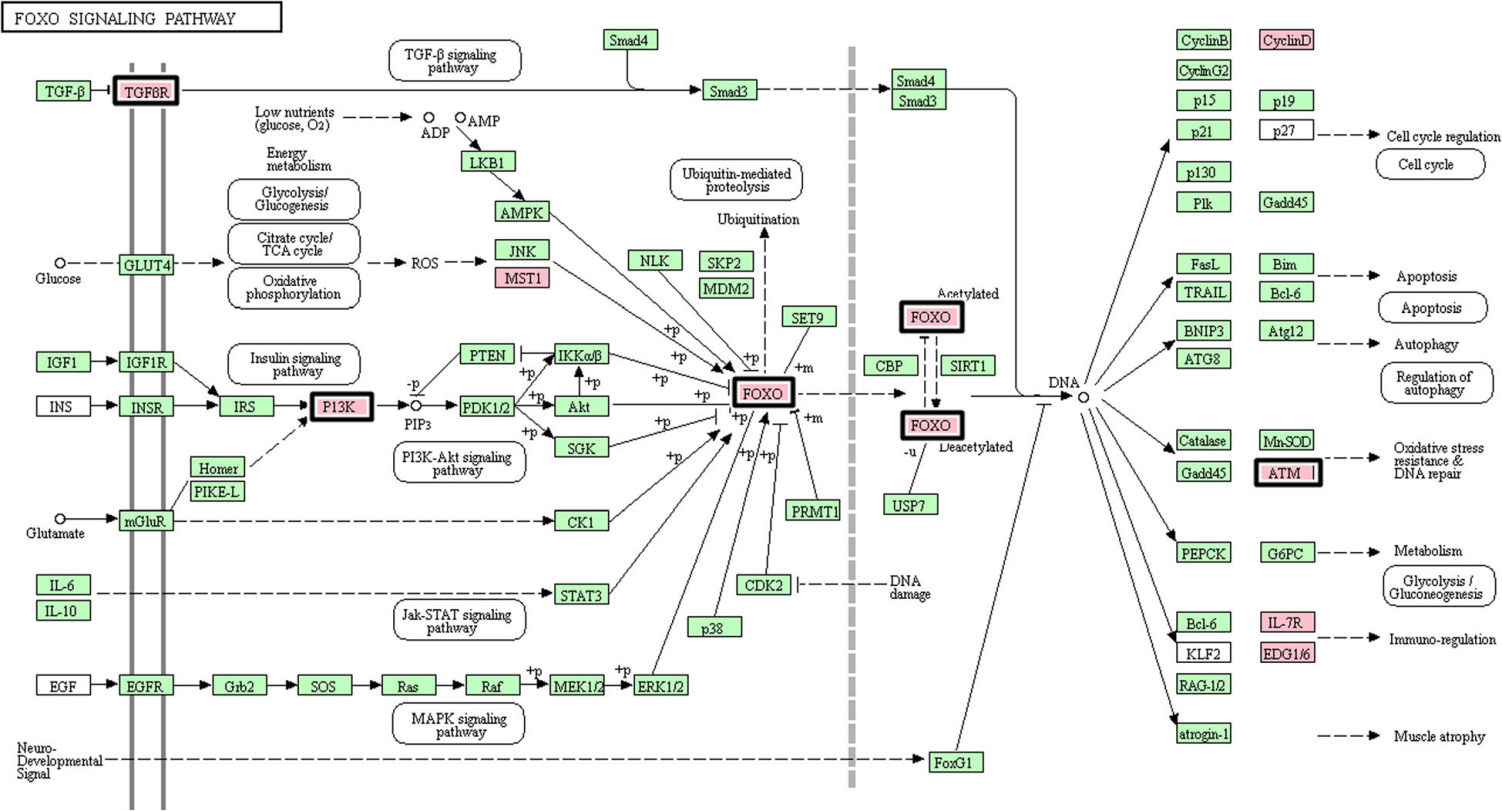
Significantly up-regulated genes during analysed training periods in Arabian horse blood that belong to the FoxO signalling pathway (KEGG ecb04068). Pathway presents the genes identified as differentially expressed (adjusted *p*-value < 0.05) between the T_2_ vs. T_3_ periods (bold highlight) and between T_0_ vs. T_3_ (pink highlight)

**Fig. 2:**
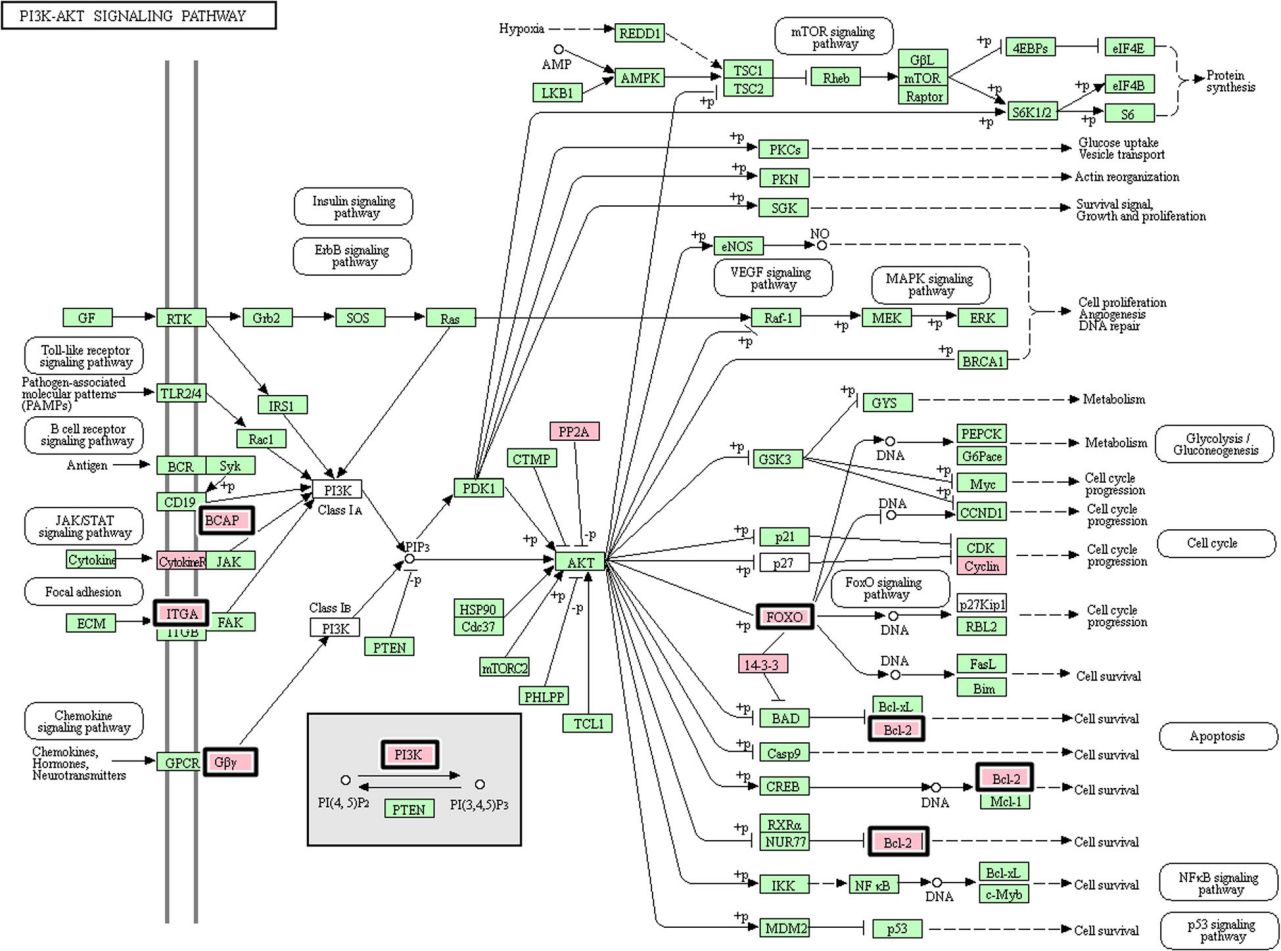
Significantly up-regulated genes during analysed training periods in Arabian horse blood that belong to the PI3K-Akt signalling pathway (KEGG ecb04151). Pathway presents the genes identified as differentially expressed (adjusted *p*-value < 0.05) between the T2 vs. T3 periods (bold highlight) and between T_0_ vs. T_3_ (pink highlight)

Pathway analysis was performed with DAVID and KEGG software using as an input a set of differentially expressed genes (fold change of at least ± 1.3; adjusted *p*-value < 0.05) between subsequent training periods: T_1_ vs. T_2_; T_2_ vs. T_3_ and T_0_ vs. T_3_. The most abundant genes up-regulated in exercise were involved in pathways that are important for the regulation of cell cycle (PI3K-Akt signalling pathway), cell communication (cAMP-dependent pathway), proliferation, differentiation and apoptosis, as well as immunity processes (Jak-STAT signalling pathway). We also observed the exercise-induced expression of genes related to regulation of the actin cytoskeleton, gluconeogenesis (FoxO signalling pathway, insulin signalling pathway), glycerophospholipid metabolism and calcium signalling. The identified genes belonged to the two most overrepresented pathways, the FoxO and PI3K-Akt signalling pathways, which are highlighted in Figs. 1 and 2. Moreover, an increase in the transcriptional activity of genes belonging to the Notch signalling pathway (*KAT2B, NCOR2, NOTCH1, NOTCH2* and *RBPJ*) was identified (Table 4). Due to the relatively low number of detected down-regulated genes, only one pathway was found to be significant, and it contained genes overexpressed at the start of the training schedule (T_1_): hypertrophic cardiomyopathy (HCM) pathway (*CACNB1, TPM1, TPM2, TTN*).

**Table 4:** The significant exercise-regulated molecular pathways identified fallowing training in Arabian horses

Pathway	Kegg number	Gene N	Gene symbol	*p*	Gene N	Gene symbol	*p*	Gene N	Gene symbol	*p*
Comparison		T_1_ vs T_2_			T_2_ vs T_3_			T_0_ vs T_3_		
Jak-STAT signaling pathway	ecb04630				4	*BCL2, IL6ST, PIK3CG, PTPN11,*	0.03	6	*BCL2, CCND2, IL6ST, IL7R, PIK3CG, PTPN11,*	0.01
PI3K-Akt signaling pathway	ecb04151				6	*BCL2, FOXO3, GNBa, ITGA4, PIK3AP1, PIK3CG,*	0.01	11	*BCL2, CCND2, FOXO3, GNB4, IL7R, ITGA4, PIK3AP1, PIK3CG, PPP2R5E, YWHAG, GNGT2*	0.001
cAMP signaling pathway	ecb04024				2	*PDE3B, PIK3CG*	0.04	7	*ATP2B, CAMK4, NFATC1, PDE3B, PDE4A, PIK3CG, PPP1R12A*	0.003
FoxO signaling pathway	ecb04068				4	*ATM, FOXO3, PIK3CG, TGFBR1,*	0.03	8	*ATM, CCND2, FOXO3, IL7R, STK4, PIK3CG, S1PR4, TGFBR1*	0.001
Glycerophospholipid metabolism	ecb00564	4	*AGPAT5, LGPAT1, GPD1, PLA2G4F*	0.001	4	*AGPAT3, AGPAT5, GPD2, LPGAT1,*	0.01	5	*AGPAT3, AGPAT5, GPD2, LPGAT1, PCYT1A*	0.002
Insulin signaling pathway	ecb04910				4	*CBL, CBLB, PDE3B, PIK3CG*	0.02	5	*CBL, FASN, CBLB, PDE3B, PIK3CG*	0.02
Calcium signaling pathway	ecb04020	2	*CYSLTR1, SLC25A4*	0.01	3	*ITPR1, ITPR2, CYSLTR1*	0.03	5	*ATP2A2, ATP2B4, CAMK4, ITPR1, ITPR2,*	0.03
Regulation of actin cytoskeleton	ecb04810	1	*MYLPF*	ns	6	*ARHGEF6, IQGAP2, ITGA4, PAK2, PIK3CG, SSH2,*	0.01	8	*ARHGEF6, IQGAP2, ITGA4, MSN, PAK2, PIK3CG, SSH2, PPP1R12A*	0.02
Notch signaling pathway	ecb04330				2	*KAT2B, RBPJ*	ns	5	*KAT2B, NCOR2, NOTCH1, NOTCH2, RBPJ*	0.001
Hypertrophic cardiomyopathy (HCM)	ecb05410	4	*CACNB1, TPM1, TPM2, TTN,*	0.03	1	*ITGA4*	ns	1	*ITGA4*	ns

*N* number of identified genes, *ns* not significant; gene symbol indicated in bold were identified as down-regulated during training periods

### Validation of results by qPCR

**Fig. 3:**
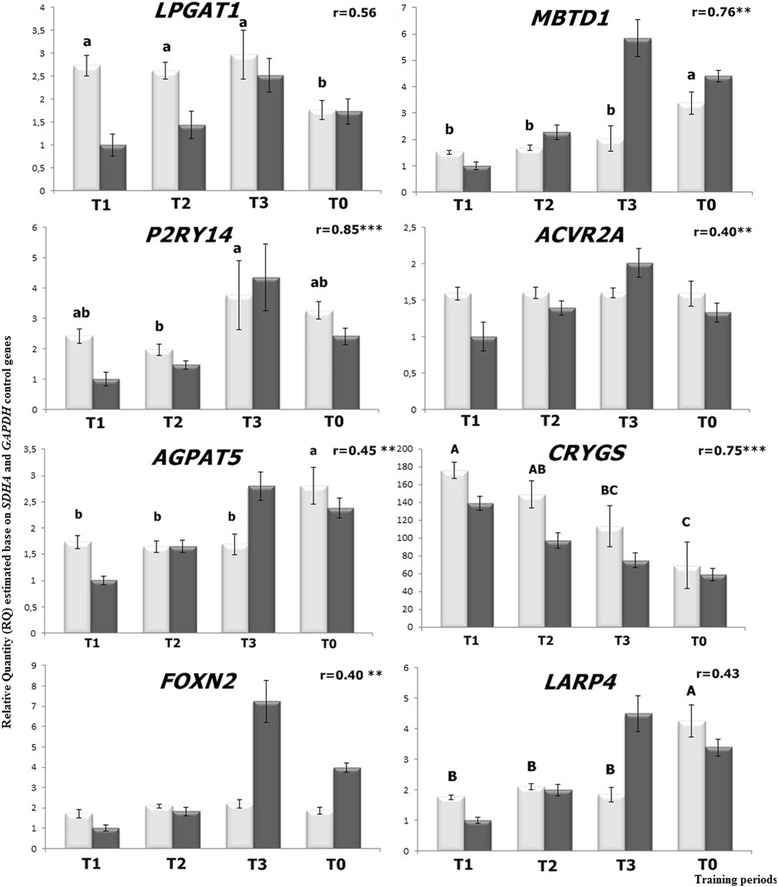
The exact gene expression levels estimated by real-time PCR (white column) and normalized counts detected by RNA-seq (grey column) for selected genes. The data are presented as mean ± standard error (training periods are T_1_: after the slow canter phase, T_2_: after the intense gallop phase, T_3_: at the end of the racing season; and T_0_ are untrained horses; the mean of Relative Quantity with different letters vary in significance with a,b: *p* < 0.05 and A,B: *p* < 0.01; r: correlation coefficient; * *p* < 0.05; ** *p* < 0.01; ***p < 0.001)

The validation of RNA-seq differential expression data was performed using a real-time PCR method for eight exercise-regulated genes. The significant positive correlation coefficients between normalized read counts (RNA-seq) and relative quantity results (real-time PCR) were observed for most of the analysed genes (Fig. 3). For six genes, significant differences in transcript levels were detected between training periods (*CRYGS; LARP4; MBTD1; AGPAT5; LPGAT1; P2RY14*). qPCR confirmed the increased expression of the *P2RY14* and *LPGAT1* genes during the training schedule when compared to untrained horses. For *LARP4* and *MBTD1*, an increase in transcript levels during training was detected (from T_1_ to T_3_ periods), but the highest expression was identified in untrained horses. On the other hand, the expression level of the *CRYGS* gene decreased from the T_1_ to T_3_ training period, which was consistent with the RNA-seq results. The differences were not identified for the *FOXN2* and *ACVR2A* genes; however, a trend similar to that of the RNA-seq data was observed (fold change values for both methods are presented in Additional file 5: Table S4). The scarce dissimilarities observed between the RNA-seq and qPCR data probably result from the incomplete annotation of the horse genome (for example, the presence of other, unknown gene splice variants).

## Discussion

Arabian horses are believed to be one of the oldest and most influential horse breeds in the world. The Arabian lineage was used to improve many breeds, such as the Thoroughbred and the Lipizzaner, and they are still contributing to the refinement of other breeds [21]. Compared to other breeds, Arabian horses are characterized by remarkable stamina and courage; thus, they dominate in endurance riding and racing. The racing performance traits in Thoroughbred horses have been widely described. On the other hand, the exact mechanisms that occur in the blood and muscles of the Arabian horses during exercise and how they are related to their skeletal muscle workload are still not well understood.

Arabians, when compared to TB, have a lower maximal rate of oxygen consumption (VO_2_max), lower running speed at VO_2_max, lower respiratory exchange ratio and higher free fatty acid consumption in substrate selection during low-intensity exercise [22]. Moreover, Arabian horses have a higher proportion of type I and IIa (oxidative) muscle fibres in comparison to Thoroughbreds, which tend to have more fibres that support ATP production via anaerobic metabolism [23–25]. These differences support the thesis that the performance traits of Thoroughbred and Arabian horses are conditioned in different ways [23, 25], but the genetic aspects of racing performance have not been established for Arabian horses.

In the present research, blood transcriptomes obtained from 18 Arabian horses were analysed using an NGS-based RNA-Seq approach. To identify the genetic basis of adaptive response to training, we compared global gene expression profiles between subsequent training periods and between two extreme points: horses in the last training phase and untrained horses. The analysis of overrepresented functional ontologies showed that the most abundant exercise-regulated transcripts formed a group of genes encoding transcription factors (34 DEGs for the T_2_ vs. T_3_ comparison and 43 DEGs for T_0_ vs. T_3_). This result was consistent with previous research performed on Thoroughbred horses before and after exercise [10]. The authors identified the differential expression of 91 transcription factors in equine blood and muscle tissues, which suggested that the identified genes are responsible for the regulation of exercise-triggered signalling pathways. The present study showed that a large number of DEGs encode proteins that are involved in nucleic acid binding, act as G-protein modulators or have kinase activity in Arabian horse blood during exercise. This result was probably an exercise-related response to restore body homeostasis through the activation and/or repression of many signalling pathways and adaptation to exercise.

During the training schedule, our results indicated a significant up-regulation of several pathways involved in cell cycle regulation, proliferation, differentiation and apoptosis (PI3K-Akt signalling and Jak-STAT signalling pathways), as well as cell-cell communication (cAMP-dependent and Notch signalling pathways) and cellular signalling (calcium signalling pathway). It has been established that exercise increases protein synthesis via activation of the PI3K-Akt signalling pathway, which regulates inter alia skeletal muscle hypertrophy [26, 27]. The PI3K-Akt acts by increasing protein synthesis though the activation of mTOR pathways and by inhibiting protein degradation through the inactivity of FOXO transcription factors [27, 28]. The present research also confirmed the significant impact of Arabian horse training on the overrepresentation of both the PI3K-Akt and FoxO signalling pathways, as represented inter alia by the differential expression of *FOXO3* (Forkhead box protein O3), *PIK3CG* (Phosphoinositide-3-kinase PI3K), *TGFBR1* (TGF-beta receptor type-1), *BCL2* (Apoptosis regulator Bcl-2) and *ATM* (Serine-protein kinase ATM).

Comparisons of the T_2_ vs. T_3_ and T_0_ vs. T_3_ training periods showed exercise-induced expression of genes belonging to the Jak-STAT and cAMP signalling pathways; this expression was especially evident between the horses at the end of the training stage and the untrained horses (T_0_ vs. T_3_). In humans, Trenerry et al. [29] indicated that intensive exercise increased the levels of proteins involved in the JAK/STAT signalling pathway (interleukin-6, platelet-derived growth factor-BB), and the authors established that the investigated proteins are essential in adaptive responses to intensive training. Furthermore, it has been proven that cAMP signalling regulates the hypertrophic response in skeletal myofibres by increasing myofibre size and initiating fibre-type conversion [30–32]. In turn, in endurance horses, training induces the activity of oxidative fibres and decreases anaerobic metabolism, which is associated with an increase in the percentage of Type I fibres in muscle tissues [33]. During subsequent training stages, we observed an increase in the transcriptional activity of the *PIK3CG* gene (belonging to phosphoinositide-3-kinases, PI3Ks), which plays a critical role in most of the identified exercise-regulated pathways. In rats, Cheng et al. [34] showed that exercise enhances the protein levels of cardiac IGFI-R/PI3K/Akt pathways as well as the Bcl-2 family, including the *BCL2* gene, which was also identified in our research. The significant function of PIK3 kinase during skeletal muscle development was confirmed in a mouse animal model [35]. According to the authors, PI3K promotes the role of myostatin (*MSTN*) in the modulation of cell survival. Based on the identified exercise-regulated pathways, it can be assumed that our results enabled detection of the molecular modifications that occur in horses and are the basis for metabolic changes in an organism’s adaptation to long-term training.

Moreover, the differential expression of genes related to metabolic processes that are critical to maintaining body homeostasis seems interesting. The results showed that the exercise-regulated genes belong to the FoxO and Insulin signalling pathways, which are essential for the glucose and glycerophospholipid metabolism pathways. In our study, eight genes from the FoxO signalling pathway were up-regulated in response to an increase in physical activity, including the *FOXO3* (Forkhead Box O3) and *TGFBR1* genes (Transforming Growth Factor, Beta Receptor 1). It has been proven that FoxO transcription factors play a key role in the regulation of energy homeostasis in muscle tissue during catabolic conditions [36]. Furthermore, FoxO3 can increase mitochondrial respiration by binding to the mitochondrial DNA-regulatory regions, and both FoxO1 and FoxO3 can regulate the two main proteolytic pathways (ubiquitin–proteasome and autophagy–lysosome pathways) [37]. The recent study performed by Sanchez [38] confirmed the significant function of FoxO factors in response to endurance training through the regulation of angiogenetic processes in muscle. The increased expression of the Forkhead family of transcription factors (FOXOs) that was observed in our research suggests that these genes also play an important role in adaptation to exercise in horses.

One of the most important groups of DEGs associated with training in Arabian horses are involved in metabolic processes, particularly in energy production (glycerophospholipid metabolism and insulin signalling pathways). Homeostasis between the utilization of glucose and lipids during intensive exercise is critical for the optimization of training efficiency. It has been shown that, in comparison to Thoroughbreds, the muscle tissue of Arabian horses is characterized by a higher proportion of slow twitch (Type I) muscle fibres [24; 25], which are characterized by a low glycogen content and a high reserve of triglycerides. The increased request of lipids during training can be confirmed by the observed up-regulation of genes belonging to the glycerophospholipid metabolism pathway in our results. The improved efficiency of fatty acid utilization was particularly evident between horses in the last training period and untrained horses, where five significant genes were related to lipids metabolism: *AGPAT3, AGPAT5, GPD2, LPGAT1,* and *PCYT1A*. Two of these genes belonged to the AGPAT family (acylglycerolphosphate acyltransferase), which catalyses an important step in triacylglycerol (TAG) synthesis and storage [39]. The glycerol-3-phosphate dehydrogenase 2 gene (*GDP2*) encodes the mitochondrial protein responsible for glycerone phosphate synthesis and is an element of glycerol degradation via the glycerol kinase pathway [40]. In turn, Choline-phosphate cytidylyltransferase A (*PCYT1A*) controls phosphatidylcholine biosynthesis, while lysophosphatidylglycerol acyltransferase 1 (*LPGAT1*) takes a part in the glycerophospholipid biosynthetic process [41].

Furthermore, our results pinpointed few transcripts that were affected by exercise and classified as part of the insulin signalling pathway (*CBL, FASN, CBLB, PDE3B, PIK3CG* – PI3Ks). Some of these genes control adipogenesis (*FASN*; fatty acid synthase), while others are considered negative regulators of many pathways, including the insulin signalling pathway (E3 ubiquitin-protein ligases: *CBL* and *CBLB*) [42]. Kim et al. [43] confirmed that endurance training increased the expression levels of several genes belonging to the insulin pathway (also PI3-kinase; Phosphoinositide 3-kinases) in rat skeletal muscles and suggested that this kind of exercise can be associated with alterations in insulin sensitivity. In 2002, the research performed on mice established that the increase in PI3-kinase activity after exercise resulted in the enhancement of insulin activity in skeletal muscles [44]. To date, the significant role of PI3Ks in insulin-mediated glucose uptake following training has been confirmed in humans and other species [45]. In Thoroughbred horses, McGivenly et al. [6] detected the up-regulation of insulin and Type II diabetes mellitus pathways after short-term exercise and proposed that these changes were transcriptional adaptations to training. Our results also highlighted the significance of molecular mechanisms for modulating glucose uptake and lipid metabolism. These mechanisms are essential for maintaining body homeostasis during long-term exercise in Arabian horses.

Regular training is associated with the increased expression of antioxidant enzymes and the activation of inflammatory cytokines [46], which are generally recognized by the organism as stress factors [4]. One of the most important adaptive responses to exercise is changes in gene expression that are responsible for metabolic modification and cell cycle progression, which ultimately result in a return to homeostasis. In Arabian endurance horses, Cappaelli et al. [20] identified four genes that had high sequence similarity to genes involved in a training-induced stress response: *IL8* (interleukin 8), *EIF4G3* (eukaryotic translation factor 4 gamma, 3), *RBBP6* (retinoblastoma binding protein 6) and *HSP90AA1* (heat shock protein). In 2010, Capomaccio et al. [12] showed the differential expression of several genes involved in inflammation in horses participating in endurance races. Global gene expression profiles of the blood were analysed in three periods (pre-, during and post-race). Among the 20 identified genes with the highest fold change, the authors detected three interleukins (*IL18; IL8; IL1R2*), three chemokines (*CXCL2; CCR2; CCL5*) and an interferon receptor 1 (*IFNAR1*). Similarly, Capomaccio et al. [11] compared the blood transcriptomes at two time points, at rest and immediately at the end of the race, and showed exercise-induced deregulation of pro-inflammatory molecules, such as *IL8, IL18, CCR2, TLR1, IL1R1, CXCL1, CCL5* and integrins (*ITGAL, ITGAM*). In the present study, we attempted to estimate the influence of long-term exercise periods on gene expression patterns in the blood of Arabian horses. A significant up-regulation of interleukins (*IL6ST; IL6R; IL7R*) and integrin (*ITGA4*) was observed. The increased transcript abundance of acute-phase response molecules confirmed that an organism recognizes long-term training as a stress factor and that these inflammatory responses play an important function in triggering downstream signalling pathways, which are critical to the maintenance of homeostasis. Thus, understanding the molecular basis of exercise-induced stress can be helpful for identifying performance markers in horses.

Application of an RNA-seq method based on next-generation sequencing allowed us to detect a panel of uncharacterized DEGs (51 genes) with a potentially important function in exercise adaptation. Some of these genes were differentially expressed in more than two training periods, and orthologue analysis showed that these genes mainly encoded transcription factors, defence/immunity proteins, and proteins that bind nucleic acids and receptors. Similar results were presented by Park et al. [10], who analysed exercise-induced changes in the blood transcriptome of TB horses and detected 56 uncharacterized genes that code for transcription factors. Our approach provided a panel of candidate genes that may be responsible for adaptive training responses in horses; these genes should be investigated in the future.

## Conclusions

Our results allowed for the identification of changes in gene expression profiles during a training schedule in Arabian horses. Based on a comparative analysis of blood transcriptomes, several exercise-regulated pathways and genes affected by exercise were detected. We pinpointed the overrepresented molecular pathways and genes that are essential for an exercise-related adaptive response for maintaining body homeostasis. The observed transcriptional activation of such genes as *LPGAT1, AGPAT5, PIK3CG, GPD2, FOXN2, FOXO3, ACVR1B* and *ACVR2A* can form the basis for further research to identify the genes that are potentially associated with racing performance in Arabian horses. Such markers may be essential for choosing a training regimen, and they can reveal what causes the differences in racing performance that are specific to various breeds.

## Additional files

Additional file 1: Figure S1. The training procedure and points of sample collection. (DOC 212 kb)

Additional file 2: Table S1. Primer sequences used in real-time qPCR validation. (DOC 40 kb)

Additional file 3: Table S2. The basic NGS data statistics for the analysed samples. (DOC 71 kb)

Additional file 4: Table S3. Identified gene orthologues with differential expression between analysed training periods. (DOC 112 kb)

Additional file 5: Table S4. The fold-change values obtained from comparisons between subsequent training periods using RNA-seq and qPCR methods. (DOC 38 kb)

## Abbreviations

DEGs: Differentially expressed genes
TB: Thoroughbred horses
PASB: Polish Arabian Stud Book
RIN: RNA integrity number
QC: Quality control
GO: Gene Ontology
VO2max: Maximal rate of oxygen consumption
KEGG: Kyoto Encyclopedia of Genes and Genomes
