# Supplementary material for: Transcriptome profiling of Arabian horse blood during training regimens
Source: BMC Genet. 2017 Apr 5;18:31. doi: 10.1186/s12863-017-0499-1 (PMC5382464; doi:10.1186/s12863-017-0499-1)
Supplement: Supplementary file 1 — The training procedure and points of sample collection. (DOC 212 kb) [file 12863_2017_499_MOESM1_ESM.doc]

S1 Text: The training procedure preparing for flat racing and points of sample collection:

The experiment was performed on 6 untrained Arabian horses (2,5 years old) and 12 Arabian horses (3 years old) introduced to the training center in September. From 12 horses during training period samples were collected at three different time-points of training procedure: after the slow canter phase (March – conditioning phase), after intense gallop phase (May - before racing season -trained horses), and at the end of the racing season (October) (Figure 1). The **training preparing for** **flat racing** was the same for each analyzed horses. The training cycle consists of five stages:

1 - during the first month (October) 2.5 years old horses are trained to be ridden;

2 – the second and third month of training - in each week during the 4-day training period two slow canter (up to 1500 m) are introduced and interspersed by walk or trot;

3 - the fourth month - the increasing of slow canters up to 5000 m still interspersed by walk and trot;

4 – the fifth and sixth month - the decreasing of canter distance (to 2500 or 3000 m);

5 – the seventh month - gallop - every week a gallop distance is extended from 200 to 1200 m.

A few days before the start, each horse should run the distance in a gallop not longer than 2000 m and not shorter than 200-400 m above the planned race distance. The training for all horses was conducted according to presented scheme and was the same for each analyzed horse. The analyzed group of animals included the same number of mares and stallions.


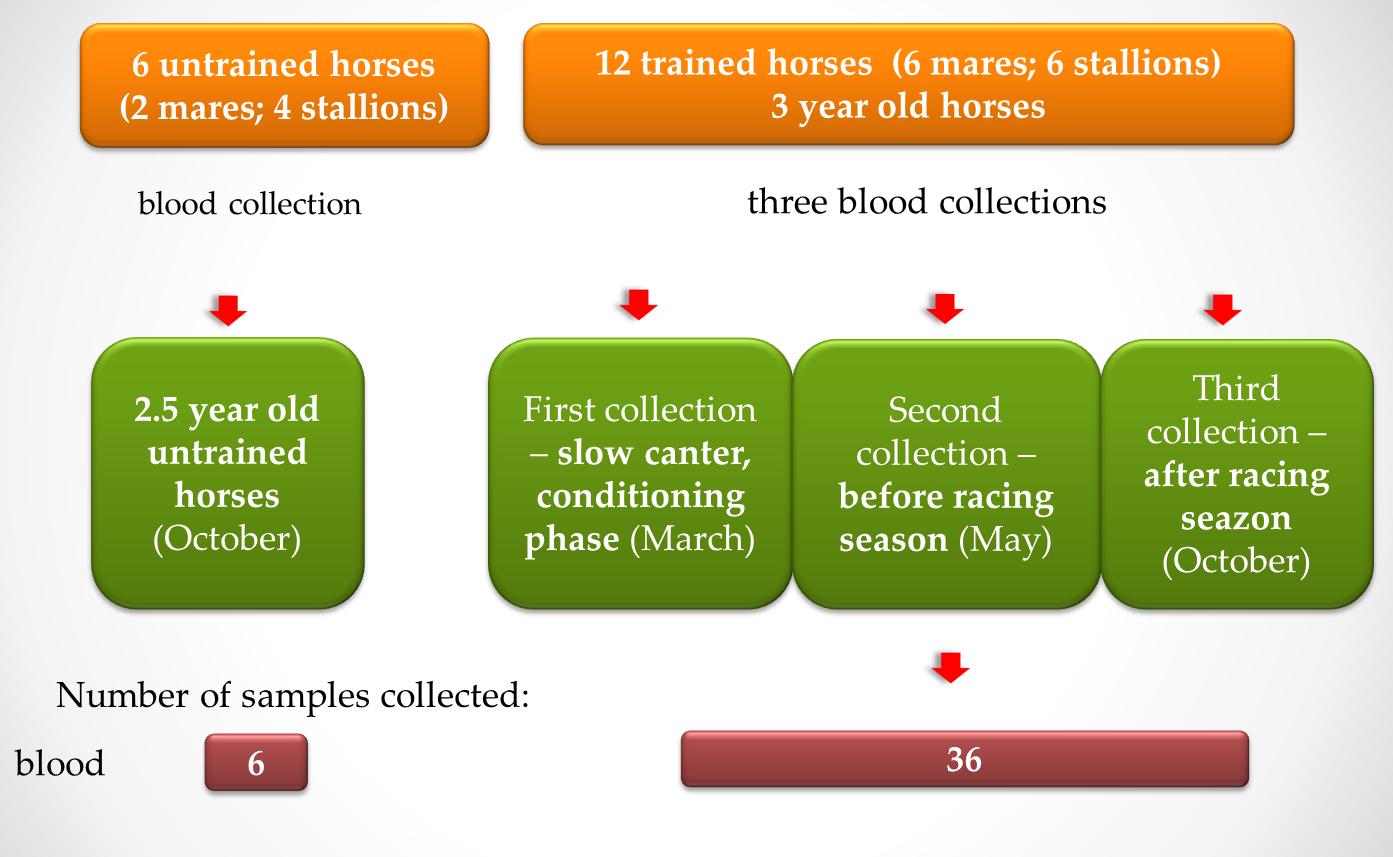


Figure 1. Time-points of sample collection during training schedule.
