# Supplementary material for: Transcriptome profiling of Arabian horse blood during training regimens
Source: BMC Genet. 2017 Apr 5;18:31. doi: 10.1186/s12863-017-0499-1 (PMC5382464; doi:10.1186/s12863-017-0499-1)
Supplement: Supplementary file 2 — Primer sequences used in real-time qPCR validation. (DOC 40 kb) [file 12863_2017_499_MOESM2_ESM.doc]

| S1 Table. Primer sequences used in real-time qPCR validation. | | | | | |
| --- | --- | --- | --- | --- | --- |
| **Gene symbol** | **Gene name** | | **Accession number** | **Sequence** | **Amplicon length** |
| ***FOXN2*** | Forkhead box N2 | | ENSECAG00000008390 | F TTTGCTACTGCACCAACAGG  R TGCAGGAGAAGCAAAAGGTT | 191 |
| ***LPGAT1*** | Lysophosphatidylglycerol acyltransferase 1 | | ENSECAG00000013187 | F TGTCACTCTGCCAAGGATTG  R TCCAAGGATCCAGGTTTGAA | 196 |
| ***AGPAT5*** | 1-acylglycerol-3-phosphate O-acyltransferase 5 | | ENSECAG00000016106 | F GATGGCTTCATGAACGTTTT  R TTCCTTCCAGCCTCAGTCAT | 194 |
| ***MBTD1*** | Mbt domain containing 1 | | ENSECAG00000022700 | F CTCCATTGCAGCACCAGTAA  R TTCTTCCCATCCATCAAAATG | 172 |
| ***ACVR2A*** | Activin receptor type-2A precursor | | ENSECAG00000000138 | F GCCATTTGAGGAGGAAATTG  R CCAGCTGATAACCTGGCTTC | 176 |
| ***P2RY14*** | Purinergic receptor P2Y, G-protein coupled, 14 | | ENSECAG00000001554 | F AGCAAACTCCTGTCGGTGAT  R GTTTGAGGCTTTGTGCCACT | 149 |
| ***CRYGS*** | Crystallin gamma S | | ENSECAG00000002645 | F CTGAACACCAGCATTGGATG  R CATGATGGAAGGGCAATCTT | 148 |
| ***LARP4*** | La ribonucleoprotein domain family member 4 | | ENSECAG00000012479 | F CAAAGCCATCGAGGACAAAT  R TCTCGTAATGGCTGCACAAG | 155 |
| ***SDHA*** | Succinate dehydrogenase complex subunit A | | ENSECAG00000000397 | F GGAAGCGTGTTACAGGAAGG  R GTAATCTTCCCTGGCGTGAG | 196 |
| ***GAPDH*** | Glyceraldehyde-3-phosphate dehydrogenase | | ENSECAG00000022051 | F TCACCAGGGCTGCTTTTAAC  R GCCTTTCCGTTGATGACAAG | 156 |
|  | |  | | | |
