# Supplementary material for: Transcriptome profiling of Arabian horse blood during training regimens
Source: BMC Genet. 2017 Apr 5;18:31. doi: 10.1186/s12863-017-0499-1 (PMC5382464; doi:10.1186/s12863-017-0499-1)
Supplement: Supplementary file 3 — The basic NGS data statistics for the analysed samples. (DOC 71 kb) [file 12863_2017_499_MOESM3_ESM.doc]

| S2 Table. The basic NGS data statistics for the analysed samples | | | | | | | | | |
| --- | --- | --- | --- | --- | --- | --- | --- | --- | --- |
| Training periods | Samples | Indexes | Pool | Number of raw reads | Number of reads after filtration | Number of discarded reeds | % of discarded | Uniquely mapped reads | Uniquely mapped reads (%) |
| T_1_ | 1 | 5 | 1 | 26730399 | 26301320 | 429079 | 1.61 | 16924163 | 64.35 |
| T_1_ | 2 | 6 | 1 | 30222483 | 29702412 | 520071 | 1.72 | 18774931 | 63.21 |
| T_1_ | 3 | 7 | 1 | 29371815 | 28886691 | 485124 | 1.65 | 18924156 | 65.51 |
| T_1_ | 4 | 8 | 1 | 26543873 | 26109331 | 434542 | 1.64 | 18779476 | 71.93 |
| T_1_ | 5 | 10 | 2 | 34747988 | 34079063 | 668925 | 1.93 | 28214132 | 82.79 |
| T_1_ | 6 | 11 | 2 | 28698865 | 28155698 | 543167 | 1.89 | 23847177 | 84.7 |
| T_1_ | 7 | 13 | 3 | 21537589 | 21155280 | 382309 | 1.78 | 17453666 | 82.5 |
| T_1_ | 8 | 14 | 2 | 30079214 | 29484441 | 594773 | 1.98 | 23288023 | 78.98 |
| T_1_ | 9 | 15 | 3 | 22043400 | 21639075 | 404325 | 1.83 | 16627493 | 76.84 |
| T_1_ | 10 | 18 | 3 | 21634746 | 21227778 | 406968 | 1.88 | 17460674 | 82.25 |
| T_1_ | 11 | 22 | 4 | 17846046 | 17342103 | 503943 | 2.82 | 7863194 | 45.34 |
| T_1_ | 12 | 27 | 4 | 16450020 | 16145701 | 304319 | 1.85 | 13327044 | 82.54 |
| T_0_ | 13 | 21 | 4 | 22642715 | 22216364 | 426351 | 1.88 | 19451158 | 87.55 |
| T_2_ | 14 | 10 | 4 | 23961657 | 23572319 | 389338 | 1.62 | 18679678 | 79.24 |
| T_2_ | 15 | 11 | 1 | 24036968 | 23633823 | 403145 | 1.68 | 19258099 | 81.49 |
| T_2_ | 16 | 5 | 2 | 23376867 | 22897075 | 479792 | 2.05 | 18653121 | 81.47 |
| T_2_ | 17 | 6 | 2 | 21537022 | 21051693 | 485329 | 2.25 | 14956654 | 71.05 |
| T_2_ | 18 | 7 | 2 | 27537740 | 27008689 | 529051 | 1.92 | 23214008 | 85.95 |
| T_2_ | 19 | 8 | 2 | 23064315 | 22626370 | 437945 | 1.90 | 19185751 | 84.79 |
| T_2_ | 20 | 25 | 1 | 26534031 | 26132187 | 401844 | 1.51 | 21340476 | 81.66 |
| T_2_ | 21 | 22 | 3 | 21248580 | 20873831 | 374749 | 1.76 | 18174014 | 87.07 |
| T_2_ | 22 | 23 | 3 | 23214099 | 22813215 | 400884 | 1.73 | 19718737 | 86.44 |
| T_2_ | 23 | 14 | 4 | 22371584 | 21985359 | 386225 | 1.73 | 19277796 | 87.68 |
| T_2_ | 24 | 15 | 4 | 17096327 | 16779324 | 317003 | 1.85 | 12227020 | 72.87 |
| T_3_ | 25 | 14 | 1 | 27327737 | 26930023 | 397714 | 1.46 | 23468846 | 87.15 |
| T_3_ | 26 | 15 | 1 | 22982835 | 22646219 | 336616 | 1.46 | 19366389 | 85.52 |
| T_3_ | 27 | 6 | 3 | 27121349 | 26592444 | 528905 | 1.95 | 23533088 | 88.5 |
| T_3_ | 28 | 7 | 3 | 24145760 | 23671814 | 473946 | 1.96 | 20286366 | 85.7 |
| T_3_ | 29 | 8 | 3 | 28492016 | 27905161 | 586855 | 2.06 | 24480621 | 87.73 |
| T_3_ | 30 | 9 | 4 | 30014784 | 29436692 | 578092 | 1.93 | 26090642 | 88.63 |
| T_3_ | 31 | 10 | 4 | 29563636 | 29007129 | 556507 | 1.88 | 25546209 | 88.07 |
| T_3_ | 32 | 11 | 4 | 24665972 | 24217526 | 448446 | 1.82 | 21405350 | 88.39 |
| T_0_ | 33 | 16 | 4 | 22921247 | 22504808 | 416439 | 1.82 | 19689350 | 87.49 |
| T_0_ | 34 | 12 | 4 | 23735032 | 23313083 | 421949 | 1.78 | 20204759 | 86.67 |
| T_0_ | 35 | 9 | 3 | 23593090 | 23162930 | 430160 | 1.82 | 20075596 | 86.67 |
| T_0_ | 36 | 10 | 3 | 23394870 | 22973911 | 420959 | 1.80 | 19902558 | 86.63 |
| T_0_ | 37 | 7 | 4 | 24288167 | 23851326 | 436841 | 1.80 | 20548297 | 86.15 |
| The number of indexes are according to TruSeq RNA Kit v2 kit (Illumina); samples were combined in 4 (1- 4) pools. | | | | | | | | | |
