# Supplementary material for: Transcriptome profiling of Arabian horse blood during training regimens
Source: BMC Genet. 2017 Apr 5;18:31. doi: 10.1186/s12863-017-0499-1 (PMC5382464; doi:10.1186/s12863-017-0499-1)
Supplement: Supplementary file 4 — Identified gene orthologues with differential expression between analysed training periods. (DOC 112 kb) [file 12863_2017_499_MOESM4_ESM.doc]

| S3 Table. Identified gene orthologues with differential expression between analysed training periods. | | | | | | | | | | |
| --- | --- | --- | --- | --- | --- | --- | --- | --- | --- | --- |
| **Gene Accesion number** | **Ortolog** | **Panther family/subfamily** | **Protein class** | | **T1 vs T2** | | **T2 vs T3** | | **T0 vs T3** | |
|  |  |  |  | | **FC** | **p** | **FC** | **p** | **FC** | **p** |
| ENSECAG00000000325 | LOC100051586 | HLA CLASS I HISTOCOMPATIBILITY ANTIGEN, A-68 ALPHA CHAIN (PTHR16675:SF174) | immunoglobulin receptor superfamily, major histocompatibility complex antigen | | -1.61 | 0.0002 |  |  |  |  |
| ENSECAG00000008721 | LOC100052638 | GRANZYME H (PTHR24271:SF15) | serine protease | | -1.84 | 0.0000 |  |  |  |  |
| ENSECAG00000002201 | RPL23A | 60S RIBOSOMAL PROTEIN L23A (PTHR11620:SF10) | ribosomal protein | | -1.43 | 0.0003 |  |  |  |  |
| ENSECAG00000015782 | Uncharacterized protein | HLA CLASS I HISTOCOMPATIBILITY ANTIGEN. A-68 ALPHA CHAIN (PTHR16675:SF174) | immunoglobulin receptor superfamily. major histocompatibility complex antigen | | -1.59 | 0.0002 |  |  |  |  |
| ENSECAG00000009368 | Uncharacterized protein | SUBFAMILY NOT NAMED (PTHR16675:SF179) | immunoglobulin receptor superfamily. major histocompatibility complex antigen | | -1.45 | 0.0003 |  |  |  |  |
| ENSECAG00000021750 | Uncharacterized protein | HLA CLASS I HISTOCOMPATIBILITY ANTIGEN. ALPHA CHAIN F (PTHR16675:SF176) | immunoglobulin receptor superfamily. major histocompatibility complex antigen | | -1.62 | 0.0002 |  |  |  |  |
| ENSECAG00000007726 | APOBEC3Z2E | DNA DC-DU-EDITING ENZYME APOBEC-3H (PTHR13857:SF25) | nucleic acid binding. deaminase | |  |  | -1.36 | 0.0006 |  |  |
| ENSECAG00000000968 | LOC100050719 | INTERFERON-INDUCED TRANSMEMBRANE PROTEIN 3 (PTHR13999:SF4) | - | |  |  | -1.39 | 0.0024 |  |  |
| ENSECAG00000002368 | LOC100059314 | SUBFAMILY NOT NAMED (PTHR11758:SF8) | ribosomal protein | |  |  | -1.44 | 0.0010 |  |  |
| ENSECAG00000017143 | LOC100060531 | HLA CLASS II HISTOCOMPATIBILITY ANTIGEN. DQ BETA 2 CHAIN (PTHR19944:SF56) | major histocompatibility complex antigen | |  |  | -1.49 | 0.0016 |  |  |
| ENSECAG00000018890 | MDP1 | MAGNESIUM-DEPENDENT PHOSPHATASE 1 (PTHR17901:SF14) | - | |  |  | -1.47 | 0.0001 |  |  |
| ENSECAG00000025060 | MYH3 | MYOSIN-3 (PTHR13140:SF427) | G-protein modulator. actin binding motor protein. cell junction protein. | |  |  | -1.41 | 0.0027 |  |  |
| ENSECAG00000020428 | RP9 | SUBFAMILY NOT NAMED (PTHR13484:SF1) | - | |  |  | -1.38 | 0.0002 |  |  |
| ENSECAG00000014226 | Uncharacterized protein | | | |  |  | -1.52 | 0.0025 |  |  |
| ENSECAG00000015782 | Uncharacterized protein | HLA CLASS I HISTOCOMPATIBILITY ANTIGEN. A-68 ALPHA CHAIN (PTHR16675:SF174) | | immunoglobulin receptor superfamily. major histocompatibility complex antigen |  |  | -1.83 | 0.0000 |  |  |
| ENSECAG00000016428 | Uncharacterized protein (Fragment) | HLA CLASS I HISTOCOMPATIBILITY ANTIGEN. A-68 ALPHA CHAIN (PTHR16675:SF174) | | immunoglobulin receptor superfamily. major histocompatibility complex antigen |  |  | -1.51 | 0.0002 |  |  |
| ENSECAG00000017490 | LIMS1 | LIM AND SENESCENT CELL ANTIGEN-LIKE-CONTAINING DOMAIN PROTEIN 1-RELATED (PTHR24210:SF11) | | structural protein. actin family cytoskeletal protein |  |  | 1.46 | 0.0291 |  |  |
| ENSECAG00000017795 | Uncharacterized protein | | | |  |  | 1.46 | 0.0368 |  |  |
| ENSECAG00000013923 | Uncharacterized protein | - | | - |  |  | 1.47 | 0.0323 |  |  |
| ENSECAG00000025018 | ZFX | ZINC FINGER X-CHROMOSOMAL PROTEIN (PTHR24387:SF200) | | KRAB box transcription factor |  |  | 1.55 | 0.0145 |  |  |
| ENSECAG00000019609 | ZNF814 | SUBFAMILY NOT NAMED (PTHR24387:SF223) | | KRAB box transcription factor |  |  | 1.40 | 0.0411 |  |  |
| ENSECAG00000021005 | AKAP11 | A-KINASE ANCHOR PROTEIN 11 (PTHR10226:SF3) | | kinase modulator |  |  | 1.45 | 0.0178 | 1.53 | 0.0110 |
| ENSECAG00000009566 | GOPC | GOLGI-ASSOCIATED PDZ AND COILED-COIL MOTIF-CONTAINING PROTEIN (PTHR16528:SF2) | | - |  |  | 1.48 | 0.0301 | 1.61 | 0.0062 |
| ENSECAG00000022544 | KMT2C | - | | - |  |  | 1.55 | 0.0330 | 1.96 | 0.0001 |
| ENSECAG00000016162 | LOC100062658 | POLYCOMB GROUP RING FINGER PROTEIN 5 (PTHR10825:SF34) | | ubiquitin-protein ligase |  |  | 1.51 | 0.0012 | 1.57 | 0.0008 |
| ENSECAG00000022703 | LY5 | LYMPHOCYTE ANTIGEN 75 (PTHR22803:SF65) | | receptor |  |  | 1.52 | 0.0254 | 1.69 | 0.0001 |
| ENSECAG00000002472 | NHSL2 | NHS-LIKE PROTEIN 2 (PTHR23039:SF2) | | - |  |  |  |  | 1.52 | 0.0150 |
| ENSECAG00000000464 | POM121C | NUCLEAR ENVELOPE PORE MEMBRANE PROTEIN POM 121-RELATED (PTHR23193:SF5) | | structural protein. RNA binding protein |  |  |  |  | 1.39 | 0.0414 |
| ENSECAG00000022168 | PRR14L | - | | - |  |  | 1.56 | 0.036518 | 1.88 | 0.0007 |
| ENSECAG00000000883 | RBM27 | RNA-BINDING PROTEIN 27 (PTHR14398:SF1) | | RNA binding protein |  |  | 1.46 | 0.018069 | 1.57 | 0.0025 |
| ENSECAG00000013174 | SRCAP | HELICASE SRCAP (PTHR10799:SF638) | | DNA helicase. helicase |  |  |  |  | 1.44 | 0.0191 |
| ENSECAG00000006365 | TAF1 | TRANSCRIPTION INITIATION FACTOR TFIID SUBUNIT 1-RELATED (PTHR13900:SF0) | | acetyltransferase. acyltransferaza. protein kinase. transcription factor. nuclease. |  |  |  |  | 1.49 | 0.0016 |
| ENSECAG00000015324 | TBL1X | F-BOX-LIKE/WD REPEAT-CONTAINING PROTEIN TBL1X-RELATED (PTHR22846:SF43) | | - |  |  | 1.41 | 0.030858 | 1.39 | 0.0198 |
| ENSECAG00000015123 | TRAPPC10 | TRAFFICKING PROTEIN PARTICLE COMPLEX SUBUNIT 10 (PTHR13251:SF3) | | - |  |  | 1.42 | 0.036518 | 1.43 | 0.0033 |
| ENSECAG00000023195 | Uncharacterized protein | E3 UBIQUITIN/ISG15 LIGASE TRIM25 (PTHR24103:SF332) | | - |  |  |  |  | 1.48 | 0.0207 |
| ENSECAG00000017861 | Uncharacterized protein | ANKYRIN REPEAT DOMAIN-CONTAINING PROTEIN 11 (PTHR24145:SF3) | | - |  |  |  |  | 1.65 | 0.0021 |
| ENSECAG00000014186 | Uncharacterized protein | - | | - |  |  |  |  | 1.65 | 0.0246 |
| ENSECAG00000013923 | Uncharacterized protein | | | |  |  |  |  | 1.59 | 0.0031 |
| ENSECAG00000017946 | Uncharacterized protein | - | | - |  |  |  |  | 1.87 | 0.0001 |
| ENSECAG00000009756 | Uncharacterized protein (Fragment) | ACYL-COENZYME A THIOESTERASE 1-RELATED (PTHR10824:SF16) | | - |  |  |  |  | 1.74 | 0.0062 |
| ENSECAG00000022043 | Uncharacterized protein (Fragment) | - | | - |  |  | 1.44 | 0.019018 | 1.67 | 0.0004 |
| ENSECAG00000011016 | Uncharacterized protein (Fragment) | INTERMEDIATE FILAMENT FAMILY ORPHAN 2 (PTHR14516:SF1) | | - |  |  |  |  | 1.45 | 0.0155 |
| ENSECAG00000014696 | Uncharacterized protein (Fragment) | INTERFERON-ACTIVABLE PROTEIN 203-RELATED (PTHR12200:SF16) | | - |  |  |  |  | 1.53 | 0.0191 |
| ENSECAG00000002280 | ZFP91 | - | | - |  |  |  |  | 1.39 | 0.0130 |
| ENSECAG00000002541 | ANP32A | ACIDIC LEUCINE-RICH NUCLEAR PHOSPHOPROTEIN 32 FAMILY MEMBER A-RELATED (PTHR11375:SF1) | | phosphatase inhibitor |  |  |  |  | -1.41 | 0.0242 |
| ENSECAG00000001669 | FTH1 | FERRITIN HEAVY CHAIN (PTHR11431:SF37) | | storage protein |  |  |  |  | -1.45 | 0.0336 |
| ENSECAG00000007359 | IL10RB | INTERLEUKIN-10 RECEPTOR SUBUNIT BETA (PTHR20859:SF50) | | type I cytokine receptor |  |  |  |  | -1.38 | 0.0002 |
| ENSECAG00000017143 | LOC100060531 | HLA CLASS II HISTOCOMPATIBILITY ANTIGEN. DQ BETA 2 CHAIN (PTHR19944:SF56) | | major histocompatibility complex antigen |  |  | -1.49 | 0.038682 | -1.66 | 0.0209 |
| ENSECAG00000009892 | MAP1LC3B | MICROTUBULE-ASSOCIATED PROTEINS 1A/1B LIGHT CHAIN 3 BETA 2 (PTHR10969:SF15) | | non-motor microtubule binding protein |  |  |  |  | -1.39 | 0.0401 |
| ENSECAG00000018890 | MDP1 | MAGNESIUM-DEPENDENT PHOSPHATASE 1 (PTHR17901:SF14) | | - |  |  | -1.47 | 0.013937 | -1.45 | 0.0486 |
| ENSECAG00000010440 | SYF2 | PRE-MRNA-SPLICING FACTOR SYF2 (PTHR13264:SF5) | | - |  |  |  |  | -1.39 | 0.0486 |
| ENSECAG00000014226 | Uncharacterized protein | | | |  |  | -1.52 | 0.047614 | -1.65 | 0.0350 |
| ENSECAG00000015782 | Uncharacterized protein | HLA CLASS I HISTOCOMPATIBILITY ANTIGEN. A-68 ALPHA CHAIN (PTHR16675:SF174) | | immunoglobulin receptor superfamily |  |  | -1.83 | 0.001258 | -1.77 | 0.0124 |
| FC (fold change) -fold change value with minus - genes down-regulated; | | | | | | | | | | |
