# Supplementary material for: Transcriptome profiling of Arabian horse blood during training regimens
Source: BMC Genet. 2017 Apr 5;18:31. doi: 10.1186/s12863-017-0499-1 (PMC5382464; doi:10.1186/s12863-017-0499-1)
Supplement: Supplementary file 5 — The fold-change values obtained from comparisons between subsequent training periods using RNA-seq and qPCR methods. (DOC 38 kb) [file 12863_2017_499_MOESM5_ESM.doc]

| S4 Table. The fold-change values obtained from comparisons between subsequent training periods using RNA-seq and qPCR methods. | | | | | | | | | | | | |
| --- | --- | --- | --- | --- | --- | --- | --- | --- | --- | --- | --- | --- |
|  | **RNA-seq results** | | | | | | **qPCR results** | | | | | |
|  | **T_1_ vs T_2_** | adj pvalue | **T_2_ vs T_3_** | adj pvalue | **T_3_ vs T_0_** | adj pvalue | **T_1_ vs T_2_** | p value | **T_2_ vs T_3_** | p value | **T_3_ vs T_0_** | p value |
| *LPGAT1* | -1.40 | ns | 1.39 | 0.01 | 1.46 | 0.02 | -1.10 | ns | 1.13 | ns | -0.59 | 0.05 |
| *MBTD1* | 1.48 | ns | 1.65 | 0.009 | 1.61 | 0.005 | 1.11 | ns | 1.20 | ns | 1.67 | 0.05 |
| *P2RY14* | 1.54 | 0.05 | 1.69 | 0.009 | -1.59 | ns | -1.23 | ns | 1.91 | 0.05 | -1.16 | ns |
| *ACVR2A* | 1.56 | 0.01 | 1.55 | 0.003 | -1.45 | ns | 1.01 | ns | 1.00 | ns | -1.10 | ns |
| *AGPAT5* | 1.55 | 0.011 | 1.60 | 0.02 | 1.52 | 0.02 | 1.05 | ns | 1.03 | ns | 1.65 | 0.05 |
| *CRYGS* | -1.39 | 0.007 | -1.43 | 0.004 | 1.47 | ns | -1.20 | ns | -1.31 | ns | -1.61 | ns |
| *FOXN2* | -1.41 | ns | 1.80 | 0.007 | 2.01 | 8.78E-06 | -1.22 | ns | 1.04 | ns | -0.84 | ns |
| *LARP4* | -1.40 | ns | 1.54 | 0.013 | 1.65 | 0.005 | 1.20 | ns | -0.87 | ns | 2.30 | 0.001 |
| adj p value are presented according to DESeq2; p value were calculated using Duncan test (ANOVA procedure ); ns- not significant | | | | | | | | | | | | |
